# Why marry early? Parental influence, agency and gendered conflict in Tanzanian marriages

**DOI:** 10.1017/ehs.2022.46

**Published:** 2022-10-21

**Authors:** Jitihada Baraka, David W Lawson, Susan B Schaffnit, Joyce Wamoyi, Mark Urassa

**Affiliations:** 1 National Institute for Medical Research, Mwanza, Tanzania; 2 Department of Anthropology, Pennsylvania State University, Pennsylvania, USA; 3 Department of Anthropology, University of California, Santa Barbara, California, USA

**Keywords:** Sexual conflict, parent–offspring conflict, child marriage, bridewealth, human behavioural ecology

## Abstract

Global health interventions increasingly target the abolishment of ‘child marriage’ (marriage under 18 years, hereafter referred to as ‘early marriage’). Guided by human behavioural ecology theory, and drawing on focus groups and in-depth interviews in an urbanising Tanzanian community where female early marriage is normative, we examine the common assumption that it is driven by the interests and coercive actions of parents and/or men. We find limited support for parent–offspring conflict. Parents often encouraged early marriages, but were motivated by the promise of social and economic security for daughters, rather than bridewealth transfers alone. Moreover, forced marriage appears rare, and adolescent girls and young women (AGYW) were active agents in the transition to marriage, sometimes marrying against parental wishes. Support for gendered conflict was stronger. AGYW were described as being lured into unstable relationships by men misrepresenting their long-term intentions. Community members voiced concerns over these marriages. Overall, early marriage appears rooted in limited options, encouraging strategic, but risky choices on the marriage market. Our results highlight plurality and context dependency in drivers of early marriage, even within a single community. We conclude that engaging with the importance of context is fundamental in forging culturally sensitive policies and programs on early marriage.

## Graphical abstract

**Figure:**
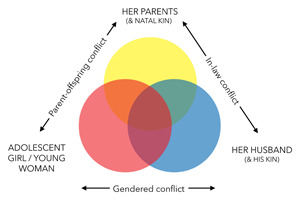

**Social media summary:** Study reveals multiple pathways to early marriage of adolescent girls and young women in Mwanza, Tanzania

## 1. Introduction

The concept of ‘child marriage’ is defined in global health and international development discourse as any marital union where at least one partner is under 18 years of age. Marriage of adolescent girls and young women (AGYW) under 18 years was historically ubiquitous, including within the past century in the global north (Dahl, 2010; Syrett, 2016), and is most common today in rural sub-Saharan Africa and South Asia (UNICEF, 2018). A global campaign to end child marriage has emerged over the last two decades, as evidenced by a dramatic increase in research (Efevbera & Bhabha, 2020), public awareness (Lawson, Lynes, Morris, & Schaffnit, 2020) and dedicated interventions, including changes to the legal age at marriage in both global south and global north nations (Arthur et al., 2018; Muthengi, Olum, & Chandra-Mouli, 2021; Reiss, 2021). This movement emerged following a century of broader attitudinal shifts towards the concept of childhood, in which children have become viewed as increasingly vulnerable, in need of protection and unprepared for adult responsibility (Dahl, 2010; Hart, 2009; Lancy, 2008; Syrett, 2016).

Outside of global health intervention, marriages under 18 years among AGYW are typically subject to equivalent or similar influences, customs and constraints as marriages that occur in the years just above this threshold. Furthermore, in settings where early marriage is normative, those who do not marry under 18 years generally marry very soon after. We therefore prefer the term ‘early marriage’, rather than child marriage, positioning our hypotheses as concerning relatively youthful transitions to marriage generally, rather than focusing on a legally defined, but otherwise arbitrary, threshold of 18 years. The term ‘child marriage’ also encourages misunderstanding, conjuring notions of predominantly prepubescent brides, while in reality, the large majority of ‘child brides’ worldwide marry during later adolescence, at 15 years or above (Lawson et al., 2020). For example, in the northern Tanzanian setting of the current study, an estimated 35% of female marriages occur under 18 years, but only 2% below 15 years (Schaffnit, Hassan, Urassa, & Lawson, 2019).

**Figure 1.**
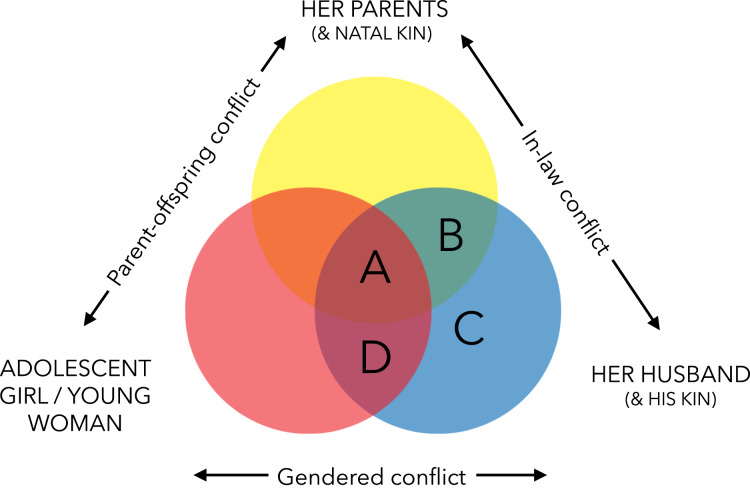
Who benefits from early marriage? Venn diagram illustrating the interests of adolescent girls and young women (AGYW), their parents and husbands following marriage. We conclude that early marriage of AGYW occurs under a range of scenarios in this setting (indicated by the letters A–D), but that early marriage always serves the interests of the husband (within the blue circle). In some of these cases, an AGYW and her parents also benefit (scenario A, where all circles overlap). In other cases, parent–offspring and/or gendered conflict are implicated, with marriage coming at a cost to the AGYW (scenario B), both the AGYW and her parents (scenario C) or just the parents (scenario D). Further complexity can be added by recognising that the wellbeing consequences and fitness consequences (i.e. production of descendants) may not overlap, such as when marriage is costly for the wellbeing of AGYW, but leads to higher reproductive success.

Marriage before 18 years has been argued to have a suite of negative consequences for AGYW, including poor sexual and reproductive health, lower educational attainment and reduced physical and mental wellbeing. Supporting this conclusion, many studies demonstrate statistical, although not necessarily causal, associations between marrying before age 18 years and a range of undesirable outcomes (Muthengi et al., 2021; Nour, 2009; Raj, 2010). With such apparent evidence of harm, we must ask why early marriage remains so prevalent, and by extension who, if anyone, benefits from early marriage (Figure 1)? Schaffnit & Lawson (2021) propose human behavioural ecology (HBE) as a theoretical framework to address these questions. HBE is committed to an exploration of context-dependent costs and benefits of alternative behavioral ‘strategies’ across and within socioecological settings. It takes an optimality approach to understanding behavioral variation, predicting that adaptive strategies will be favoured by natural selection, even at a cost to individual or societal wellbeing (Nettle, Gibson, Lawson, & Sear, 2013; Winterhalder & Smith, 2000). This framework can be used to generate hypotheses about the rationality of decision making among available options, while also identifying conflicts of interest between family members that may ultimately drive inequalities and restrict women's autonomy. As a subfield of anthropology, HBE also draws heavily on ethnographic insight, adding further depth and understanding of how cultural context shapes behaviour (Gibson & Lawson, 2015; Tucker & Rende Taylor, 2007).

In global health discourse, AGYW are frequently viewed as passive victims of early marriage, coerced directly or indirectly by patriarchal norms, into marital arrangements that do not serve their best interests. Illustrating this point, the terms ‘forced marriage’ and ‘child marriage’ are often used synonymously, and treated as legally equivalent (Al Akash & Chalmiers, 2021; Schaffnit, Wamoyi, Urassa, Dardoumpa, & Lawson, 2020). In a HBE framework, this perspective can be specified as early marriage being a product of a parent–offspring conflict (Trivers, 1974) and/or a sexual/gendered conflict of interests between men and women (Borgerhoff Mulder & Rauch, 2009). In the former scenario, a girl's parents may benefit from a higher bridewealth payment (or a lower dowry) when a daughter marries young because she has higher value on the marriage market and/or because it reduces the economic burden of continuing to provide for their daughter. This leads parents to arrange or promote early marriages, despite their costs to daughters. Marrying daughters early, for example, may benefit the wider family by enabling parents to generate required capital to then marry off their sons (Schaffnit, Hassan, et al. 2019; see also Mace 1996).

In the latter scenario, which we refer to as gendered conflict, and which is not mutually exclusive with parent–offspring conflict, early marriage occurs because it benefits men, despite costs to women. In this scenario, men may prefer younger brides because they are deemed more attractive, are likely to bear more children and/or because their junior age makes them relatively more subservient (Lawson, Schaffnit, Hassan, & Urassa, 2021). When each gender has a distinct optimum for a behaviour, such as the wife's age at marriage or the magnitude of a spousal age gap, we can expect the evolution of strategies to manipulate one another and gain the upper hand. In the context of marriage, this may take the form of men or women misrepresenting intentions during courtship and/or the unexpected desertion of partners after marriage at the benefit of one gender and a cost to the other (Borgerhoff Mulder & Rauch, 2009).

We recently tested predictions from these models using quantitative data on marriage partner preferences, bridewealth, marital duration and multiple dimensions of a women's wellbeing and reproductive success in the Mwanza region of northern Tanzania (Lawson, Schaffnit, Kilgallen, et al., 2021; Schaffnit, Hassan, et al., 2019; Schaffnit, Urassa, & Lawson, 2019). Consistent with parent–offspring conflict, bridewealth was larger for younger brides. However, a large majority of women self-report choosing their own husband, and marriage under 18 years had mixed and overall equivocal relationships with wellbeing. In fact, married adolescent girls had higher decision-making autonomy than their unmarried counterparts, consistent with wives having more power within a household unit than a daughter (or a granddaughter or foster child) of a household head. Likewise, at all ages of marriage, women primarily reported that marriage advanced their social status in the community. These patterns may make early marriage desirable to AGYW. Education was lower among those married early, but community members reported strong taboos against marrying school girls, suggesting that school dropout generally precedes marriages, rather than marriage taking girls out of school (Schaffnit, Hassan, et al., 2019). Consistent with gendered conflict, women report marrying somewhat older men than they would ideally prefer, but relatively larger husband-older spousal age gap was not predictive of marital stability or women's self-reported autonomy in decision making, self-reported experience of depression or reproductive success (Lawson, Schaffnit, Kilgallen et al., 2021).

In summary, while parents of AGYW and the men they marry may benefit from early female marriage, we found limited indication that it was costly to AGYW, at least when considered among locally available alternatives (i.e. in contrast to AGYW who married later or not at all within the same community). Reproductive success was also highest for those marrying at ages 15–17 years, indicating that inclusive fitness of daughters, if not necessarily their wellbeing, is maximised by early marriage (Schaffnit, Hassan, et al., 2019). These observations suggest that we must turn to alternative explanations of early marriage that acknowledge AGYW's agency in entering early marriages and their reasons for desiring and/or accepting these marriages. This is not to argue that early marriage is ideal, but rather that it may represent a tolerable scenario compared with locally feasible alternatives when wider norms restrict women's opportunities for success beyond marriage. This may be the case when marriage provides opportunities, such as emancipation from demands of a natal family, increased community respect, and/or greater reproductive success. More generally early marriage may be favoured when the alternative of remaining unmarried or delaying marriage fails to shield adolescents from risks to wellbeing such as early pregnancy, increased likelihood of raising children outside of marriage (which may lower paternal investment) and amplified exposure to sexually transmitted infections (Schaffnit et al., 2020). These conditions may characterise contexts where sex and childbirth outside of marriage are common, transactional sex is widespread, and where broader gender norms stifle social and economic independence of women, e.g. when unmarried adult women have difficulty sourcing their own income or obtaining the respect of community members. In the language of HBE, early marriage may therefore present a ‘best of a bad job’ strategy in which AGYW make the best of the cards they have been dealt (Schaffnit & Lawson, 2021).

Yet, there are also good reasons to be sceptical of these conclusions. First, self-reported autonomy in decision-making, including in the choice of marriage partners, may be both impacted by social desirably bias and obscure more subtle forms of parental influence, e.g. daughters may ‘choose’ their partners, but choices may be heavily influenced by the forecast benefits of parental approval or costs of parental disapproval. Second, reported associations, or lack thereof, between age at marriage and wellbeing, like most studies of early marriage, are vulnerable to confounding since we were limited in our ability to adjust for potentially distinct background characteristics of those that marry relatively early or late. Third, our analyses looked at the associations between age at first marriage and current wellbeing in a sample spanning a large age range. This method restricts our ability to assess trajectories of wellbeing for women, which may potentially involve short-term yet recoverable costs obscured by longer-term resilience. Fourth, while quantitative analyses are fundamental at establishing generalised patterns, they can come at a cost of masking potential plurality in the drivers and consequences of early marriage. For example, unless analyses are stratified by appropriate subgroups, aggregated trends may give the false impression of null relationships between age at marriage and wellbeing, while in reality early marriage may be beneficial for some and costly for others.

To address these shortcomings, and more generally triangulate our assessment of the drivers of early marriage, here we turn to qualitative data. This work builds on complementary work in which we explored understandings of risk and opportunity during female adolescence (Schaffnit et al., 2020) and community knowledge and understanding of the global health concept of child marriage (Schaffnit et al., 2021). Here, we focus specifically on interrogating the relevance of parent–offspring conflict and gendered conflict to early marriage. This is achieved via analysis of focus groups and in-depth interviews with AGYW, young men and parents of AGYW close to the typical age of marriage.

## 2. Methods

### 2.1. Study context

**Figure 2.**
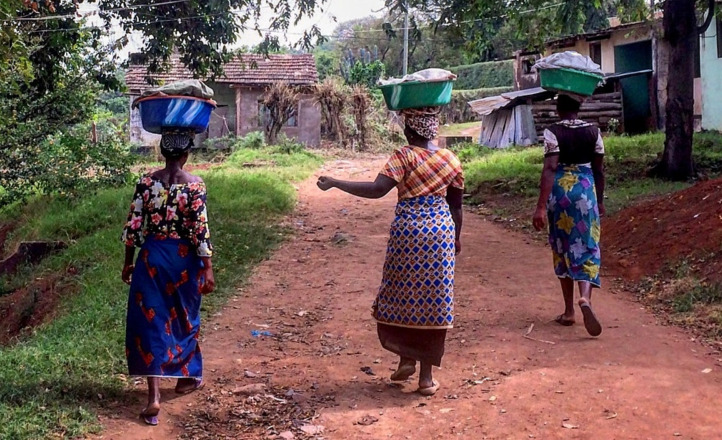
Women walking in the Mwanza region, Tanzania. We estimate that approximately one third of adolescent girls and young women marry under 18 years in the study area, meeting the definition of ‘child marriage’. Image credit: Susan Schaffnit.

Data were collected in a semi-urban town in Mwanza Region located within a Health and Demographic Surveillance Site, managed by the National Institute for Medical Research (NIMR; Kishamawe et al., 2015; Figure 2). Most residents belong to the Sukuma ethnic group. The Sukuma were traditionally agropastoralists (Wijsen & Tanner, 2002) and still commonly are in rural areas, while in more urban areas have diverse livelihoods. Men and, increasingly, women work as petty traders, labourers or skilled workers, or participate in small business. Adolescent girls commonly conduct domestic work in their natal household, particularly in later adolescence when time spent on chores increases substantially (Hedges, Sear, Todd, Urassa, & Lawson, 2018). Formal education for girls has increased in the population with urbanisation, now matching or even exceeding boys’ education (Hedges et al. 2018). While schooling reduces farm and household work, this is truer for boys; girls who attend school still contribute substantially to domestic chores, effectively taking on a ‘double shift’ of school and domestic work, reducing their time allocation to leisure (Hedges et al. 2018).

Virginity is not a pre-requisite for marriage and past research in the community has estimated that median ages at first sex are around 16 years for girls, with many AGYW sexually active prior to marriage (Marston et al., 2009). As a consequence, especially in light of limited sexual and reproductive health services, childbearing before or outside of marriage is common. Divorce is not exceptional and may be initiated by either partner, and is typically followed quickly by remarriage, at least for women of childbearing age (Boerma et al., 2002). Transactional sex outside of marriage occurs and is embedded in wider cultural and gendered expectations that men should provide for women's material needs in sexual relationships and that women should reciprocate by means of sex (Wamoyi et al., 2019). A double standard applies to sexual behaviour; a woman's reputation may be damaged if she is seen as promiscuous, while men's reputation is generally enhanced by greater sexual activity. Extra-marital affairs are typically blamed on women not men (Wight et al., 2006). Polygynous marriage is permitted, but monogamous marriage is much more common. Marriages vary in formality, but almost always involve cohabitation. In formal marriages, a ceremony occurs and bridewealth is typically transferred from a man to his parents-in-law. Bridewealth may be composed of cows, goats, other livestock, money and/or other gifts. In informal marriages, without ceremony, a delayed bridewealth or ‘compensation’ transfer may occur depending on approval from the family and availability of resources. Informal marriages are more common in circumstances when bridewealth cannot be afforded and/or the couple marry without parental approval. At the time of data collection, marriages under age 18 years were legal in Tanzania, but became illegal two months later, coming at the end of a several-year court decision (Schaffnit et al., 2020).

Sukuma families traditionally follow patrilineal inheritance and patrilocal post-marital residence, but norms are flexible (Wijsen & Tanner, 2002), and influenced by recent urbanisation, which is increasing neolocal residence. Notions of male authority and women's subordination in marriage are reinforced and reflected in traditional Sukuma songs (Masele & Lakshmanan, 2021). Attitudinal surveys suggest diversity in men's beliefs, but men typically support male authority in decision-making, including with respect to dissolving unhappy marriages and adding cowives. Men appear relatively more supportive of women taking on wage-labour and believe in the value of girls’ education (Lawson, Schaffnit, Kilgallen et al., 2021). Intimate partner violence in the wider Mwanza region is common (around 60% of women report experiencing some form of intimate partner violence in their lifetime), and under many circumstances it is deemed socially acceptable (Abramsky et al., 2019; Kapiga et al., 2017; see also Kilgallen et al., 2021).

### 2.2. Study design and data collection

Data collection took place from June to August 2019. A total of 13 focus group discussions (FGDs), each with six to eight participants, and 26 in-depth interviews (IDIs) were conducted. IDI and FGD participants were purposively sampled. The targeted participants for the FGDs and IDIs were parents (mothers and fathers) of AGYW of marriable age, AGYW aged between 15 and 18 years old, women aged 19–24 years and men aged 20–30 years (Table 1). The ages of the girls, young women and men were selected to include those who were likely to recently have been or soon would be married. FGDs captured attitudes surrounding early marriage and female adolescence broadly shared within the community, while IDIs allowed a deeper exploration of complex and personal views of the same topics. FGDs were structured around three vignettes based on amalgamated stories of adolescence collected by the authors (for details see Schaffnit et al., 2020). Each vignette told the story of a fictional local girl and were designed to understand opportunities available to adolescent girls when they are not in school, and explore situations in which either a girl wishes to marry but her parents disagree, or parents wish their daughter to marry, but the daughter disagrees. IDIs were more conversational and explored four main themes: (1) participants’ knowledge of ‘child marriage’, (2) how they heard of ‘child marriage’, (3) their experience of the phenomena, and (4) their opinion on the theme (for details see Schaffnit et al., 2021).

**Table 1.** The number of in-depth interviews and focus group discussion with each targeted group

Participants		IDI (*n* = 26)	FGD (*n* = 13)
Fathers of young girls		4	2
Mothers of young girls		3	2
15–18 year old girls	Married	3	3
	Unmarried	3	
19–24 year old women	Married	3	3
	Unmarried	4	
20–30 year old men	Married	3	3
	Unmarried	3	

FGD participants were recruited by FGD facilitators along with an employee of NIMR. A list of potential participants for FGDs were identified from the HDSS based on age, sex and marital status. Research assistants contacted potential participants and then used snowballing techniques to recruit further participants from the neighbourhood in which the target lived. Facilitators recruited IDI participants from FGD participants based on marital status in the case of girls and young adults. All discussions were conducted by Tanzanian social scientists of the same gender as the participant(s) in Swahili or Sukuma. During recruitment, participants were led through a consent process culminating in collection of their signature. Participants were given a hard copy of a study information sheet. Parental/guardian consent was collected during recruitment of minors and the minor provided assent at the time of the FGD/IDI. FGDs and IDIs were audio-recorded, transcribed and later on translated to English.

This study was granted ethical approval by the Tanzanian National Institute of Medical Research Lake Zone Institutional Review Board (MR/53/100/595), the National Ethical Review Committee (NIMR/HQ/R.8a/Vol.IX/3104) and the University of California Santa Barbara Human Subjects Committee (2-18-0993). Informed consent was also gained at the community level via a presentation of the study objectives, requirements and projected outputs to village leadership.

### 2.3. Data analysis

A hybrid approach was used to analyse data and draw out themes and sub-themes, combining both an inductive and deductive approach (Fereday & Muir-Cochrane, 2006). In an initial inductive phase, the lead author reviewed transcripts of all FDGs and IDIs to become familiar with the discussion and drew up a codebook of dominant narratives. This then was supplemented by a deductive phrase in which transcripts were explored purposely with an expanded codebook addressing any indications of parent–offspring conflict or gendered conflict, by flagging descriptions of early marriage as having bad/good/mixed consequences for AGYW, men and parents respectively. Another categorisation focused on agreement and disagreement in perspectives on child marriage between genders and generations, however few participants commented on how views on early marriage contrasted between groups of community members. Excerpts of transcripts were managed and analysed in NVIVO 12 (Wong & Li Ping, 2008), with the first and second author making judgements on the most illustrative and representative quotations to present. While only one author read the transcripts for this particular analysis, this limitation was countered by co-author familiarity with the transcripts from prior analyses of the data, with co-authors previously reading and coding material in related analyses of community opinions on female adolescence and marital transitions (Schaffnit et al., 2020, 2021).

## 3. Results

Below we summarise our findings, identifying four main themes: perceived benefits of early marriage to parents; the agency of AGYW in the marriage process; perceived risks of early marriage; and the role of men in encouraging AGYW to marry. Throughout, we identify the source of commentary (AGYW, fathers, mothers, young men), but emphasise that, overall, we found largely similar perspectives on early marriage across each of these groups.

### 3.1. Parents see economic benefits to early marriage

Participants recognised that early marriage occurred in the community and generally thought that these marriages were illegal, even though the legality of marriage under 18 years was under national debate during data collection (becoming illegal shortly after):

*Some are getting married while they don't have even breasts they just grow while in the marriages … They consider it normal, she has grown up. (IDI, 15–18 year old girl)*

*Currently there are laws, for example when you let a child under the age of 18 get married, there are laws prohibiting that because these children will drop from school and the government does not like it when children under the age of 18 get into relationships with adults. (IDI 19–24 year old woman)*

Nevertheless, parents were described as encouraging of early marriage when it was viewed as the best way to counteract economic insecurity and secure a stable future for their daughter. This sentiment was expressed both by parents directly, and by younger men and in AGYW impressions of parental beliefs. In this sense, early marriages to financially secure men were often deemed appropriate.

*... her parents may advise her to get married because they have seen some benefit to the man marrying their daughter. You see, maybe they have seen he is financially capable. (IDI 19–24 year old woman)*

*So those parents who have a high convincing power … when they see that the man who is courting their daughter has a lot money they [the parents] convince the daughter to get married. (IDI 20–30 year old man)*

Marriage to a financially secure husband, before or after 18 years, was generally understood to benefit daughters to some extent, but the immediate payment of bridewealth and the potential for the daughter's relationship to also relieve financial insecurity at home was also mentioned.

*To be married at younger age might be because of parent's influence, that they need money, cow, [etc.] … so they think of a better way is to wed their daughter at younger age so as to get the bridewealth and meet their needs at home. (IDI, father)*

*Yet others think that the money or things that they get from bridewealth will help them and so they think it is better for the girls to be married. (IDI, 20–30 year old man)*

*Maybe you find the condition at home is bad, therefore the parents persuade their daughter to get married and parents advise her to save some money to assist them [the parents]. (IDI, 19–24 year old woman)*

In such cases, there was an understanding that early marriage could go against daughters’ best interests, while relieving hardship for the wider family.

*I think parents, especially those with low financial capabilities, usually see that when a daughter grows up, then they should marry her off so that the bride price will help them in their different needs at home and so that is the benefit that they look at, but they do not consider the negative effects that will come to the child in future. (IDI, 15–18 year old girl)*

Both mothers and fathers may be encouraging of early marriage. While men rely on their authority over the household, close bonds between daughters and mothers mean that mothers may be especially convincing.

*I can say that the mother has more convincing power, because I have heard some who tell their children to get married early. (IDI, 20–30 year old man)*

Beyond economic concerns, adolescent misbehaviour was mentioned by parents as a motivator for early marriage, with stories of girls not sleeping at home and dropping out of school against the wishes of parents. In these circumstances, parents may feel that marriage is the best choice.

*There are some [girls] who stop listening and respecting their parents; they do crazy things and lose respect completely and so to avoid embarrassment for the parents … [the] parent finds it better for the child to go and get married. (IDI, mother)*

### 3.2. Adolescent girls and young women have agency in marriage decisions

Participants didn't only identify the benefits of marriage from parents’ perspectives. Rather, many discussed the ways that early marriage may be desirable to AGYW themselves, often owing to a lack of feasible alternative opportunities, especially when continued education was no longer possible. This could be because of a lack of resources to continue schooling or because a girl had lost interest in school. In these scenarios, agency in the decision to marry is clear.

*... some are leaving home to work at the small restaurants as they are tired of school as well as staying at home and decide to get married. (IDI, 15–18 year old girl)*

*I asked her [a married girl under 18 years] if she was not still in school … she said she had finished primary school and was just at home, so she thought it would be better to get married. (IDI, 20–30 year old man)*

*Nowadays we see [young] people are being married but people don't care … unless it is a student. Then people might take it into consideration or the parents might make follow-up [to find a daughter who eloped]. (IDI, 19–24 year old woman)*

Not continuing with education was not the only factor that could limit alternatives to marriage. Pregnancy before or outside of marriage also shifted the options available to AGYW, and could make otherwise unappealing marriages appropriate.

*I didn't decide [to marry], but it was pregnancy … So, when the pregnancy came first I said to myself even if I don't get married I will just stay at home to become an unmarried matured woman. It is better I tolerate this man and settle. (IDI, 15–18 year old girl)*

Likewise, difficulties at home, typically linked to poverty, were described as motivating early marriage as a pathway to escape hardships.

*For me, I was living with my stepfather and not my biological father. My mother got sick and she [was] paralyzed. My stepfather gave up taking care of me and my mother as well. I had nothing to do other than staying at home, and later I got a child while at home and my partner took me away. (IDI, 19–24 year old woman)*

Alternatively, rather than escape such hardships, daughters may be drawn to marriage because it offers potential to support their wider family. Here, marriage may not necessarily be in the best interests of a girl, but nevertheless represents a strategic choice on her part. One participant shared his perspective that AGYW act to convince their parents of the benefits of early marriage.

*Let us now assume that, because a child is bringing money home and the family is poor, she tries to convince her mother that she needs to be married so that she can help the family. So, parents, you need to agree that even though is not what you planned for [your daughter], [she must marry] because of the hardships at home. (IDI, 20–30 year old man)*

### 3.3. Early marriage entails risks, especially when marriages are unstable

While early marriage may typically involve female choice in this community, it was also considered a risky option. Three main factors were frequently mentioned as important risks to early marriage: (a) risk of difficulty in childbirth, (b) risk of intimate partner violence and (c) the risk of marriage being unstable, ending in divorce and constraining future alternatives for girls and young women. The first two of these risks however were not limited to married AGYW, because sex and relationships occurred before and outside of marriage (i.e. AGYW may get pregnant or experience intimate partner violence without being married). Potential harms of early childbirth were mentioned by both men and women.

*If she gets married, even giving birth will be a problem for her; she could die or fall ill at that age. (FGD, mothers)*

*So, the major side effect that affects most of these girls who get married below the age of 18 is the safety of their lives during child birth … She may lose a lot of blood because her organs are not well developed to handle child birth. (IDI, 20–30 year old man)*

Some of the girls, women and parents also agreed that early marriage of AGYW can lead to intimate partner violence, which was often discussed alongside stories of the marriage ending in separation.

*You find that most girls who run away from home to get married, their marriages do not last. What they get from the marriage is beatings and those are the common stories that we hear. (FGD, 15–18 year old girl)*

*The marriage was too much for me; the man I married was a dictator and he verbally insulted me and mistreated me. I had enough so I went to my sister. (IDI, 19–24 year old woman)*

Marriages were also deemed unstable because the girl may not be mentally prepared for the realities of married life.

*... she goes there at a young age and with no prior preparations and that is why these childhood marriages don't last for long. (FGD, father)*

*A 16-year-old cannot live with a man to create good values, and most of the children we see who get married younger, come back home after a few days because she goes into her new family, meets her in-laws, it's hard to deal with such situations as a child. (FGD, mothers)*

Failed marriages were viewed as costly because a girl loses opportunities for financial stability, but also because she may be left pregnant or with children but with little support from the father.

*There is a friend of mine here who got married around that age but when she did the man abandoned her; I don't even know where she is now. The man impregnated her, left her with two kids and fled. (IDI, 19–24 year old woman)*

This could lead to girls regretting the decision to marry early, and in some cases, frustration at their parents allowing the marriage to take place.

*[AGYW who marry early] even lack basic needs and regret why they got married. They blame their parents for allowing them to get married. (IDI, 15–18 year old girl)*

### 3.4. Men lure adolescent girls and young women into early marriages

All categories of participant noted the tendency of men to lure girls into costly unstable early marriages. Girls could be attracted to these men because of interest in sexual activity, immediate financial benefits of the relationship through material and cash gifts or because of unrealised promises of providing financial security or relief from hardships at home. Participants described such scenarios among AGYW, both in general terms when talking about the dangers of adolescence, and for specific AGYW in their community:

*... as soon as they reach puberty they are off the rails. And so, if a young man like that meets a girl like this one who is 16, has gone through puberty and does not really understand what she is being taught at school, she could be easily convinced to get married. (FGD, father)*

*He kicked her out while she was pregnant when he was the one who lured her away from her home. From what I heard, the girl left and later gave birth to her child. I don't know what went on from there but they are no longer together. (IDI, 20–30 year old man)*

Parents were particularly wary of daughters being seduced by men into unfavourable marriages, and women's stories made clear that they often involved elopements and a lack of parental approval:

*Honestly, I think us girls are usually carried away by the good times in the beginning. When he is soothing you and giving you presents, you become content and feel like you are where you should be. But what you end up facing is problem after problem and tears that run day and night. When you start crying all the time and going through suffering when no one at home really knows where you are and the man who made you elope has abandoned you, then you remember what your parents told you and you start regretting. (FGD, 19–24- year-old woman)*

One father, saw changes in the law as a pathway to preventing his daughter being lured into marriage by other men before she was mature:

*When it comes to that, I will have to use my authority as a father; the marriage is not happening and if the man tries to force [the marriage to occur], I will tell him that the law exists and I could point him towards the laws because he is destroying my child who is not yet at an age where she can get married. (FGD, father)*

These relationships could eventually lead to school dropout, if the girl was to become pregnant and/or marry. However, schooling itself was also seen as increasing risk of a girl being tempted into a relationship with a man. This could happen if she was unhappy at home, went to school hungry and/or because the school was a far distance away, meaning girls may have to rely on men for transport.

*It is very far away so the fare is not cheap, and there is a motorcycle driver who used to take her, maybe he even gives her money for a few things … at times he does not even take her to school, he takes her to his place and they spend the day there. (IDI, 20–30 year old man)*

*That is why I'm saying maybe she was not happy with life at home, she went to school with difficulty too and so when she met the young man and he enticed her. She felt like it would be better to be married instead of continuing to live the life she lived at home. (FGD, father)*

*The school's long distance contributes a lot and a girl will be tricked into getting married, by being promised money for food, being taken to school in the morning and brought back home. (FGD, mother)*

## 4. Discussion

### 4.1. Parental influence on early marriage

Our study sheds new light on the role of parents in early marriage. We previously established, via a quantitative survey of the same community, that the overwhelming majority of married women stated that they chose their own partner without help from kin, regardless of age at marriage. In conjunction with mixed and largely equivocal relationships between age at marriage and various measures of wellbeing and reproductive success, this led us to conclude that parental coercion of daughters into marriage was rare, and that by extension, an evolutionary parent–offspring conflict is unlikely to drive early marriage in this setting (Schaffnit, Hassan, et al., 2019). Our findings here broadly support this conclusion, but also make clear that parents are nevertheless encouraging of early marriage under particular conditions, and it is likely that such influence shapes the choices of AGYW. Crude dichotomies of forced or arranged marriages vs. marriages entered ‘freely’ by girls, while common in the literature, are therefore restrictive. These categories eschew the opportunity to examine more subtle forms of parental influence, including the repercussions of parental approval/disapproval of marriage partners (see also Agey, Crippen, Wells, & Upreti, under review).

It is also clear that parental influence is not always driven by parents seeking selfish gain at the expense of their daughters. Some participants spoke of cases of parents encouraging early marriages at a potential harm to AGYW. However, parents generally seemed more concerned with navigating pathways to their daughters future economic and social security. This supports accounts of parental influence in other cultural settings. For example, Archambault (2011) observed that among Maasai families, parents viewed early marriage as a means of providing for their daughters’ future economic stability when investments in schooling were not possible. This contradicted the messaging of external development and health agencies, which suggested that parents married girls for selfish aims and because they lacked an appreciation for school.

Parent–offspring conflict in an evolutionary or economic sense occurs when a behaviour benefits parents at the cost to offspring or vice-versa. Yet, a conflict of interests cannot be assumed from superficial behavioral observations of disagreement. Indeed, parents may be encouraging daughters to do what is in their daughters’ best interests, and it is not beyond reason that, via their greater life experience, that they know better about what makes a suitable life partner. That said, neither parental encouragement or approval are prerequisites for marriage; elopements – which by definition take place without parental support – were mentioned by many of our participants. Furthermore, the account of AGYW sometimes convincing their parents that an early marriage is the best option *for her family* via economic support, is illuminating. This scenario ostensibly presents an inverse conflict of *selfless* rather than selfish actors; the parent is unwilling to compromise the wellbeing of thier daughter (and so needs convincing), while the daughter wants to support her wider family by pursuing a marriage that may or may not be in her best interests. Of course, a daughter putting her wider family before her own needs may actually ultimately be in her fitness interests via kin selection, but the parents’ resistance here is puzzling for selfish actor theories of behaviour. It is also inherently recognisable as a common dynamic of human family relationships. For any parent, putting one own's interests above that of a child is not likely to be an easy decision, and requires overcoming strong drives to safeguard their children's wellbeing.

### 4.2. Female agency in early marriage

It is clear that AGYW are typically agentic in the transition to marriage, often choosing to marry early and sometimes eloping against parental will. We caution here that agency is not a dichotomous trait (see also Lokot 2021, 2022) and that agency can exist even when constrained by limited options, social pressure and lack of information. Our conclusion is echoed by a growing body of research documenting evidence of female agency in marital transitions before 18 years across a variety of settings (Al Akash & Chalmiers, 2021; Boyden, Pankhurst, & Tafere, 2012; Knox, 2017; Stark, 2018; Syrett, 2016), and runs counter to a widespread view, held by both the general public of global north nations (Lawson et al., 2020) and global heath frameworks (Al Akash & Chalmiers, 2021; Schaffnit et al., 2020), that all or most ‘child marriages’ are, by definition, forced marriages. Evolutionary anthropology has a well-documented history of downplaying female agency in mating and marriage (Hrdy, 1981), which has been addressed by research documenting women's strategic behaviours to navigate conflicts of interest with men and wider kin (e.g. Scelza, 2013). In parallel, we argue that global health is also often guilty of positioning women and children as primarily passive victims of patriarchy, limiting discussions of agency and strategic behaviour (Lokot et al., 2021; Miedema et al., 2020). In our experience, the notion that early marriage may occur because a girl chooses to marry and furthermore that it may be in her best interests, *given her feasible alternatives*, is often met with considerable resistance from those campaigning to end child marriage.

This tension can be resolved by grappling with the reality that an ideal notion of ‘childhood as a sanctuary’, free from risk and responsibly, is simply unachievable in many contexts, especially in the face of poverty (Hart, 2009). In many settings, childhood and adolescence are defined by substantial contributions to domestic and subsistence labour (Hedges et al., 2018), widespread exposure to risky sexual behaviour (Wamoyi et al., 2019) and wider gender norms dictating that, beyond marriage, there are simply few avenues for AGYW to gain social and economic security or community respect. From this perspective, early marriage as a rational choice is readily understandable and, by extension, focus is redirected from blaming culture to identifying and addressing structural constraints that limit women's options (see also Pot, 2019). In this light, interventions to ‘empower’ AGYW with agency may do little to delay age at marriage, and making ‘child marriage’ illegal, without changing the wider constraints faced by AGYW, may counterintuitively reinforce disadvantage by further reducing their options. In contrast, focusing on alleviating financial hardship, reducing incentives for transactional sex, and improving access to reproductive health services, for example, may do more to both alleviate risk exposure during childhood and adolescence, and make early marriage less desirable to AGYW and their kin (Schaffnit et al., 2020).

### 4.3. Gendered conflict in early marriage

Community members recognised that early marriages were sometimes driven by men ‘tricking’ or ‘luring’ girls into unstable marriages. In these cases, the initial economic or social benefits of the relationship give way to arguments, intimate partner violence and ultimately separation or abandonment. Such marriages were described as particularly risky for AGYW if they also were accompanied by pregnancy and child birth, limiting their future options and increasing the costs to their parents of having a daughter return home. These scenarios are consistent with at least a subset of early marriages being driven by gendered conflict that can bring harm to AGYW (and her parents), but at a benefit to the men they marry. Our findings also underscore that freedom of choice, in any domain, does not guaranteed that ‘good decisions’ are made, and that early marriage may be harmful not because of limited agency per se, but rather because decisions are made in the face of considerable risk and uncertainty about their consequences.

The notion of early marriages being driven by gendered conflict, but nevertheless involving female choice, has received relatively little attention in global health studies of ‘child marriage’. This is probably a direct extension of assuming limited female agency in the marriage process, and overemphasising the role of arranged and/or forced marriage as a generalised explanation for early marriage. In our initial research on this topic we also neglected this possibility, in part because we found only limited evidence that early marriage disadvantages female well-being (Schaffnit, Hassan, et al., 2019). There are three main reasons the costs of such early marriages may go underappreciated in quantitative analysis. First, ‘trick marriages’ may be only a subset of early marriages and consequently any negative impact is washed out by other neutral or positive relationships between early marriage and wellbeing. Second, if unions are very short term, their incidence may go unreported in participant surveys of marriage histories. Indeed, what constitutes a ‘marriage’ is not always clear in the context of customary marriage, with some unions perhaps better conceptualised as short-term cohabitating partnerships (see also Muthengi et al., 2022). Third, while such marriages might have costs, women may also be resilient with wellbeing rebounding in later years (such that wellbeing at the time of surveys is not associated with age at marriage). This may be particularly true for areas like the study community where high prevalence and cultural acceptance of divorce lessen the long-term consequences of suboptimal marriages.

Parents were aware of the apparent dangers of these unstable marriages and spoke of strategic behaviours as pathways to preventing them, such as appealing to national laws regulating early marriage. This observation illustrates that it is inadequate to view early marriage as a product of patriarchal norms that place a girl in opposition to wider society at large. Instead, greater understanding can be achieved by identifying the costs and benefits of early marriage for different actors influencing the marriage – including the AGYW herself, her spouse, her parents and wider networks of matrilineal and patrilineal kin. Each actor may attempt to influence not only the AGYW directly, but may also develop strategies to limit the influence of other actors. In this context, we observe that parents have selfish interests that encourage certain forms of early marriage (e.g. those involving favourable marriage payments), while also acting to discourage types of marriage deemed harmful to daughters but beneficial to prospective husbands (e.g. marriages to financially unstable men). More broadly, in this light, bridewealth transfers, which are also generally framed as harmful to women, can be understood as a parental strategy to ensure an honest signal of man's intention to invest in the marriage and of his ability to provide financially (see also Akurugu, Dery, & Bata, 2022 for a wider discussion of the role of bridewealth). Supporting this perspective, the absence of bridewealth payments is predictive of a higher risk of marital dissolution in this community (Lawson, Schaffnit, Kilgallen et al., 2021).

## 5. Conclusion

Our results speak to a plurality in the drivers of marital transitions, even within a single community, delimiting at least three distinct scenarios. Figure 1 provides a simplified visual representation of these scenarios, tentatively identifying the consequences respectively for AGYW, their parents and prospective spouses. We emphasise that the boundaries between these scenarios are clearer cut in hindsight, because the transition to marriage involves an unavoidable degree of risk and uncertainty. Furthermore, as we highlight below, complexity can be added by recognising that fitness and wellbeing consequences of a marriage for each actor may not always overlap. This distinction is important because, as an evolutionary approach to behaviour uniquely predicts, behavioural traditions may persist across time because of their positive impact on the production of descendants, even if there is an apparent cost to wellbeing (Gibson & Lawson 2015).

First, some AGYW, we suggest the majority, marry early in order to achieve relative social and economic security, and do so with encouragement and support from their parents. We suggest these marriages fall within both the wellbeing and fitness interests of AGYW, their parents and the husband (category A in Figure 1). Such marriages may represent the best available option for AGYW when situated among locally feasible alternatives, and are likely to be common when parents (or caretakers) lack resources for extended education of their daughters, and when prevailing cultural norms and structural constraints mean that marriage is more likely to bring social and economic capital than delayed marriage. They may also be common when AGYW are pregnant, or have already had a child, and wish to marry the father of their child to increase the likelihood of paternal investment. In such cases, early marriage may not be best outcome for AGYW in an ideal world, but nevertheless a rational decision when contextualised among feasible options.

Secondly, some AGYW marry early to support their natal kin, but at a cost to their own wellbeing. In a subset of these marriages, marriages will be also be costly to the fitness of daughters, while benefiting the wellbeing and fitness of her parents and the husband (category B in Figure 1). If this is the case, then such marriages reflect parents winning a true evolutionary parent–offspring conflict with their daughter, and are thus more likely to require coercion or forced marriage. Since both our quantitative and qualitative findings indicate that forced marriage is rare, we believe this subset of marriages is presently not very common in this study population. In a second subset, early marriages are costly to AGYW's wellbeing, but nevertheless remains fitness maximising (therefore in category A in Figure 1 with respect to fitness, but in category B with respect to wellbeing). Fitness benefits may be accrued to AGYW by increasing the likelihood her siblings reproduce successfully, such as when bridewealth brings in capital to help parents marry off her brothers. This helps to explain why daughters may actively choose such marriages. Indeed, as we have illustrated, early marriages may be favoured by daughters knowingly putting their own individual needs second to those of their wider family.

In our third scenario, early marriages are driven by attraction of AGYW to men offering immediate or promised economic and social benefits, but who ultimately fail to provide stability and security for women in the long term. These marriages are unlikely to serve the fitness or wellbeing interests of AGYW or her parents, but benefit men (category C in Figure 1). Such marriages frequently end in separation and are retroactively labelled bad decisions by AGYW. Parents of daughters appear to be particularly wary of such marriages. Without parental approval, these marriages often require elopement, and consequently lack bridewealth (although a later compensatory transfer may occur), further undermining the stability of the marriage. We believe this scenario is currently underappreciated by both the anthropological and the wider global health literature on early marriage.

There may also be marriages which fail to fit inside these categorisations. This may include cases where marriage can be understood as the daughter winning a parent–offspring or intergenerational conflict (scenario D in Figure 1). Here, parents (or an AGYW's caretakers) may prefer a daughter to remain unmarried so that they can benefit from her domestic labour, while early marriage provides an AGYW an opportunity to emancipate herself from such duties (Schaffnit & Lawson 2021). This scenario was not documented in the present analysis, but has been deemed important in others settings, and similar logic has been applied to the timing of female reproduction (Moya & Sear 2014). Considering Figure 1 as a whole also makes explicit that our framework has so far assumed that early marriage of AGYW universally serves the wellbeing and fitness interests of men. This assumption should be interrogated in future research, especially with respect to short-term partnerships, which are relatively understudied. Furthermore, it is clear that the concepts of parent–offspring conflict and gendered conflict may require elaboration to further consider what happens when the strategic interests of an AGYW's parents and husbands do not align. Our results, for example, indicate a prospective ‘in-law conflict’ categorises situations wherein parents act to prevent their daughters being ‘tricked’ into undesirable marriages that only benefit men (category C). Recognising this neglected axis could also lead to novel programmatic alternatives; instead of conceiving of parental influence over marriages as only negative, policy makers might consider their potential protective role in discouraging harmful relationships.

Qualitative methods have proved useful in delineating these modalities of early marriage, which may be obscured in quantitative analyses. Moving beyond our study context, we also emphasise that the applicability of parent–offspring conflict and gendered conflict models to early marriage will vary at the socioecological level. Contextual factors that may shape the form and consequences of early marriage include typical ages of marriage, sex and child birth, the feasibility of divorce and remarriage, the presence and direction of marriage payments and the degree of parental involvement in matchmaking (Schaffnit & Lawson, 2021). It is encouraging then that there are signs that global health professionals are beginning to engage more seriously with issues of context and variation, as evidenced by a recent collection of papers devoted to exploring ‘the diversity and complexity of child marriage’ (Muthengi et al., 2021), and to explore more nuanced conceptualisations of agency in transitions to early marriage (Lokot et al. 2021, 2022). Human behavioural ecology has much to contribute to these discussions, by providing a complementary theoretical framework that centralises both formal modelling of context-dependency and ethnographic observation.

## Research transparency and reproducibility

Data supporting our findings are directly reported in the manuscript (i.e. anonymised quotations from focus group and in-depth interview transcripts). Full transcripts are not suitable for being made publicly available due to the sensitive nature of the content and the possibility of participant identification.
